# The Importance of Hydrogen Bonded Networks in the Dynamic Heterogeneity of Deep Eutectic Solvents

**DOI:** 10.1021/acs.jpcb.5c02468

**Published:** 2025-06-16

**Authors:** Allison Stettler, Gary A. Baker, G. J. Blanchard

**Affiliations:** † Department of Chemistry, 3078 Michigan State University, East Lansing, Michigan 48824-1322, United States; ‡ Department of Chemistry, 14716 University of Missouri-Columbia, Columbia, Missouri 65211, United States

## Abstract

We have examined the composition-dependent rotational diffusion dynamics of the chromophores perylene (neutral), oxazine 725 (cationic) and disodium fluorescein (anionic) in a series of choline chloride/1,3-propanediol (ChCl/PD) deep eutectic solvents (DESs). We find that the rotational diffusion dynamics of all chromophores do not correlate with the bulk viscosities of any of these ChCl/PD DES compositions, consistent with dynamic heterogeneity in all cases. Comparison with other DES systems—choline chloride/ethylene glycol (ChCl/EG) and choline chloride/glycerol (ChCl/Gly)—enables evaluation of the influence of hydrogen bond donor (HBD) structure and hydroxyl group density. This comparison highlights the critical role of hydrogen-bonded networks in these DESs and underscores the pivotal function of ChCl in disrupting these networks at ca. 15 mol % ChCl across all three DES systems. Taken collectively, these data point to the constituent structural factors that mediate the properties of DESs on local and bulk scales.

## Graphical abstract

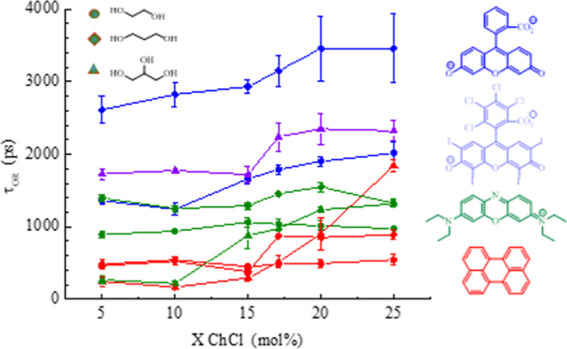

## Introduction

In recent years, the search for green alternatives to preexisting processes has become a major global issue. Many key industrial processes rely on volatile organic compounds (VOCs) that are flammable and toxic to both humans and the environment.^1–3^ These characteristics have led to the search for environmentally friendly alternatives, including ionic liquids (ILs) and deep eutectic solvents (DESs). While ILs show promise, recent studies have shown that, in addition to cost concerns, certain ILs are toxic and are therefore unsuitable as universal substitutes for current systems.^4–6^ In response to this, a significant research effort has focused on the potential implementation of DESs in academic and commercial industrial processes. DESs are biodegradable, less toxic, and display vastly reduced flammability and volatility in comparison to the ubiquitous VOCs.^7–10^ In addition, components of DESs are typically inexpensive, and their synthesis requires few, if any, purification steps, proceeding with 100% atom efficiency in a “solventless” manner.^9,11,12^ For example, the canonical DES, reline, is composed of choline chloride (ChCl) (a common nutritional supplement for cattle) and urea (a common component of fertilizer) in a 1:2 (ChCl/urea) molar ratio.^8,13^ The applications of DESs include, but are not limited to, biomass processing, redox battery production, metals processing, CO_2_ gas capture, electroplating, fossil fuel desulfurization, micellar chemistry, and biocatalysis.^8,10,14–20^

In this work, we use the term “DES” broadly. We recognize that its use has been the subject of some debate based on the range of mixture compositions that warrant the label. For example, the work of Agieienko and Buchner presents evidence that the “true” eutectic composition of ChCl/EG might be 1:4.85, rather than the originally reported 1:2.^21^ In the interest of brevity, consistency with current literature, and in recognition of the evolving technical and practical definitions of DESs, we use the terms mixture(s) and DES(s) interchangeably when referring to the binary systems described herein, depending on context and readability. This is done with the full knowledge that the classification of these materials, and the distinction between “true” DESs, eutectic mixtures, and molten salt mixtures remains under debate. However, from a pragmatic standpoint, the systems we consider here exhibit all the useful characteristics of a prototypical DES.

In general, DESs comprise mixtures of two or more constituents that, upon mixing, display a melting point significantly lower than any of its constituents individually. It has been hypothesized that the formation of these liquid phase mixtures is primarily driven by hydrogen bonding interactions between constituents with contributions from electrostatic, van der Waals, and charge transfer interactions.^8,10,14^ These solvents are relatively new discoveries, having been formally reported in 2003.^13^ Given the central role of hydrogen bonding in their formation, the constituents of a DES are typically categorized as hydrogen bond acceptors (HBAs) and hydrogen bond donors (HBDs). The diverse array of suitable components has prompted the delineation of DESs into five principal categories, so far:

- type I, a quaternary ammonium salt and a nonhydrated metal chloride (e.g., choline chloride + ZnCl_2_),
- type II, a quaternary ammonium salt and a hydrated metal chloride (e.g., choline chloride + CrCl_3_·6H_2_O),
- type III (the most commonly studied type), a quaternary ammonium salt and an organic HBD, such as an amide, carboxylic acid, alcohol, polyol, sugar, sugar alcohol, or amino acid (e.g., choline chloride + ethylene glycol),
- type IV, a metal chloride and a hydrogen bond donor (e.g., ZnCl_2_ + urea); and
- type V, which comprises nonionic components composed of neutral molecular HBAs and HBDs (e.g., lactic acid + glucose).^8,10,22^ These systems are often referred to as “natural DESs” (NADESs) when the components are naturally occurring.

Given the multitude of possible component combinations, it is clear that DESs represent a highly versatile tool for a wide range of applications in both research and industry. This abundance of potential formulations allows DESs to be specifically tailored to suit countless processes. However, to efficiently design DESs to meet targeted needs, a thorough understanding of both their bulk and local properties is essential. This, in turn, requires insight into the structural organization and molecular interactions within DES environments.

As a relatively new class of solvent, DESs remain poorly characterized in terms of their structural organization and dynamics. Their typically high viscosity further complicates efforts to investigate these properties, posing challenges for experimental and computational characterization alike. In addition to their high viscosity, DESs have been shown in previous studies to exhibit significant dynamic heterogeneity, with limited (or inconsistent) evidence of static heterogeneity across a wide temperature range. This complexity makes it challenging to unravel the interdependence between bulk properties and molecular-scale processes.^23–28^ Dynamic heterogeneity in these mixtures manifests as a nonlinear dependence of probe rotational diffusion dynamics on DES bulk viscosity and temperature. This phenomenon is interpreted as the decoupling of diffusive motion from changes in temperature and bulk viscosity.^29^ Several studies detected varying degrees of decoupling, depending on the length scale examined.^22,29–31^ Because the behavior of DES microenvironments differs significantly from that of the bulk, it is challenging to analyze, understand, and ultimately predict the properties for systems other than those extensively examined, or to forecast performance in specific applications.

The effect of temperature on the extent of decoupling for specific DESs having fixed composition ratios has been investigated more extensively, but the component ratio-dependence of DESs on viscosity decoupling is, to our knowledge, relatively unexplored.^27,29,30,32^ In a previous study, we determined that changing the molar ratios in the ChCl/ethylene glycol (ChCl/EG) system gave rise to rotational diffusion dynamics of neutral and cationic fluorescent probes that showed only limited composition-dependent changes that appeared to be significantly decoupled from the bulk DES viscosity. The negatively charged fluorescent probe used in that same study interacted more prominently with the DES system, underscoring the importance of anionic species in both mediating and sensing dynamics and organization in the ChCl/EG system.^33^ In a subsequent study, we investigated ChCl/glycerol (ChCl/Gly) mixtures to assess the generality of our previous findings. Our data point to a decoupling of dynamics over different length scales for all fluorescent probes, with effects significantly more pronounced in the ChCl/Gly system than those previously observed in ChCl/EG.^34^ These findings imply a high degree of dynamic heterogeneity in the bulk system and, based on the rotational diffusion dynamics of the fluorescent probes, the local environment(s) experienced by the chromophores appear to be isolated from the structural units within the DES that are responsible for the observed bulk properties.^34^ Investigating the ChCl/1,3-propanediol (ChCl/PD) system is a logical progression, given that the viscosity of 1,3-propanediol (53 cP at 20 °C)^35^ lies between that of ethylene glycol (18 cP at 20 °C) and glycerol (1400 cP at 20 °C). Investigating 1,3-propanediol alongside ethylene glycol and glycerol offers valuable insights into how the number and positioning of hydroxyl (−OH) groups, as well as the aliphatic spacer between them, influence local environments within these systems. Ethylene glycol, a 1,2-diol, has two hydroxyl groups on adjacent carbon atoms, leading to distinct hydrogen-bonding patterns compared to 1,3-propanediol which has hydroxyl groups separated by an additional methylene (−CH_2_−) unit. Meanwhile, glycerol, with its three hydroxyl groups, adds complexity by enabling extensive hydrogen bonding, leading to significant self-interaction.

We present an investigation of the ChCl/PD DES system, utilizing charged (disodium fluorescein, DSF^2–^; oxazine 725, Ox725^1+^), and neutral (perylene) fluorescent probes. Employing time-correlated single photon counting (TCSPC) time-domain fluorescence spectroscopy, we examine the rotational diffusion dynamics of these probes as a function of the ChCl/PD molar ratio. We then compare these data with our earlier work on the ChCl/EG and ChCl/Gly systems. The purpose of this work is to probe the generality of the previously observed viscosity decoupling in common type III DESs and to better understand the role of the HBD structure, particularly the number and spacing of −OH groups, in mediating the decoupling of dynamics over different length scales. The specific structures of the fluorescent probes and DES constituents are shown in Figure 1.

**Figure 1:**
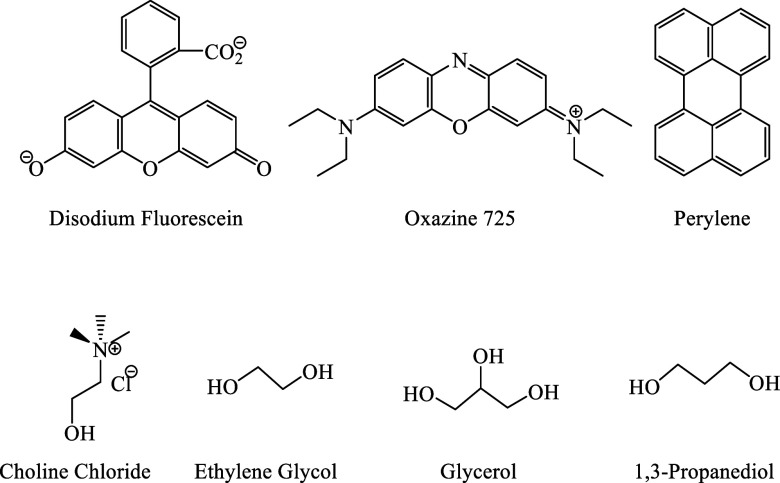
(top) Structures of chromophores used in this work. (bottom) Structures of choline chloride (HBA) and HBD constituents of DES mixtures.

## Materials and Methods

Experimental details on the synthesis of the ChCl/EG and ChCl/Gly DES systems are available in previous reports and are not repeated here.^33,34^

### Preparation of ChCl/PD DESs

Choline chloride (BioUltra, ≥99.0%; catalog no. 26978) and 1,3-propanediol (98%; catalog no. P50404) were purchased from MilliporeSigma and used as received. DESs were prepared by mixing choline chloride (ChCl) and 1,3-propanediol (PD) at appropriate mass ratios to yield samples with nominal ChCl molar percentages of 5%, 10%, 15%, 17.1%, 20%, 25%, 28.5%, and 33.3%, corresponding approximately to ChCl/PD molar ratios of 1:19, 1:9, 1:5.67, 1:4.85, 1:4, 1:3, 1:2.5, and 1:2, respectively. We note that the latter two samples remained metastable (supercooled) liquids at room temperature before eventually solidifying and were therefore excluded from further study. Exact component masses are provided in Table S1 of the Supporting Information. The specified amounts of ChCl and PD were weighed to a precision of ±0.1 mg on a Mettler Toledo NewClassic MF, model MS104*S*/03 precision analytical balance into oven-dried 250 mL round-bottom flasks. The mixtures were then subjected to rotary evaporation at 80 °C for 30–60 min at an average rotation speed of 100 rpm. The resulting homogeneous fluids were transferred into rigorously cleaned, oven-dried, and prelabeled 40 mL EPA vials, capped with PTFE-faced silicone rubber septa and equipped with PTFE-coated stirring bars. Each DES sample was further dried under vacuum at 70 °C overnight on a Schlenk line while stirring.

The chromophores used were oxazine 725 (Ox725, Exciton), perylene (Sigma-Aldrich), and disodium fluorescein (DSF, Sigma-Aldrich), and all were used as received. Chromophore stock solutions of 10^–4^ M in ethanol were used for incorporation of the chromophores into the DESs. Aliquots of these solutions were added to cuvettes in and evaporated to dryness to ensure complete removal of ethanol prior to experimentation. DES mixtures were added to the dried cuvette under an inert atmosphere and were capped. These solutions were stirred for ∼5 min. Final chromophore concentrations were ∼10^–6^ M for all DES systems reported here.

### Time-Correlated Single Photon Counting

The time-correlated single photon counting (TCSPC) instrument used here has been described in previous works^36^ and only a general description is given here. The source laser is a passively mode-locked, diode-pumped Nd/YVO_4_ laser (Spectra Physics Vanguard), which produces 13 ps pulses at a rate of 80 MHz at 1064 nm. The average power outputs of the second and third harmonic (532 and 355 nm, respectively) are 2.5 W with the same pulse duration and repetition rate as the fundamental. This pump laser excites two synchronously pumped cavity dumped dye lasers (Coherent 701-3, 7220 cavity dumpers and 7200 cavity dumper drivers). The dye laser pumped at the third harmonic of the source laser was operated with Stilbene 420 laser dye (exciton) at 435 nm (ca. 10 mW average power at 4 MHz repetition rate, 5 ps pulses) for excitation of perylene and disodium fluorescein. The dye laser excited by the second harmonic of the source laser was operated with Rhodamine 6G laser dye at 630 nm (ca. 20 mW average power at 4 MHz repetition rate, 5 ps pulses) for excitation of Ox725. The vertically polarized dye laser pulses excited the sample with less than 1 mW average power for all measurements. Fluorescence from the sample was collected perpendicular to the excitation axis using a 40× reflective microscope objective (Ealing) and split using a polarization-selective cube beam splitter (Newport Corp.). Based on the geometry of the collection volume and the concentration of the chromophores used here, we estimate that ca. 5000 chromophores are present in the collection volume at any given time, and over the course of the acquisition of a fluorescence lifetime (τ_fl_ = 3.5 ns, 50,000 max counts), ∼17 million molecules are responsible for the experimental fluorescence decay. The signal from each arm was directed to two subtractive double monochromators (Spectral Products CM 112) equipped with microchannel plate photomultiplier (MCP-PMT) detectors (Hamamatsu R3809U-50). The outputs from detectors are connected to the TCSPC detection electronics (Becker & Hickl SPC-132). The detector reference channel (Becker & Hickl PHD-400-N photodiode) originates from a fraction of the dye laser pulse train. The TCSPC electronics are controlled by software written in-house using LabVIEW software, and the response function for this system is approximately 35–40 ps fwhm. Based on a typical count rate for data collection (∼10^3^ counts/s in each channel), an emission event is recorded for ∼1 of every 2000 excitation pulses.

## Results and Discussion

As noted above, the focus of this work is on the role of HBD hydroxyl group spacing and number per molecule in determining local organization in DESs, and on how the local and bulk properties of these systems differ. These systems are known to exhibit dynamic heterogeneity, and for that reason the means we use to evaluate them must be sensitive to molecular interactions on a length scale commensurate with or shorter than the characteristic domain size(s) of the heterogeneities in the systems. Given that previous studies have detected heterogeneities in these systems in the nanoscale range, we use the rotational diffusion dynamics of charged and neutral fluorophores for this purpose. The extraction of rotational diffusion information from polarized time-domain emission decays is a well-established process, with the orientational anisotropy decay function, *R*(*t*), originating from the polarized time-resolved emission decay data (eq 1)­

1 $$R\left(t\right)=\frac{I_{∥}\left(t\right)-I_{⊥}\left(t\right)}{I_{∥}\left(t\right)+2I_{⊥}\left(t\right)}$$

The functional form of the *R*(*t*) decay contains the physical and chemical information on interest. *R*(*t*), in theory, can contain up to five exponential decays but in most cases either one or two exponential decays are observed, depending on the ellipsoidal shape of the rotor and the relative orientations of the chromophore’s absorption and emission transition dipole moments.^37^ For all the data reported here, *R*(*t*) is observed to decay as a single exponential, and the time constant of the decay, τ_OR_, is related to system properties through the modified Debye–Stokes–Einstein (DSE) eq (eq 2)^38–40^

2 $$\tau_{\text{OR}}=\frac{1}{6D_{R}}=\left(\frac{Vf}{Sk_{B}}\right)\left(\frac{\eta }{T}\right)$$

where *D*_R_ is the rotational diffusion constant, η is the bulk viscosity of the medium, *V* is the hydrodynamic volume of the reorienting entity,^41^ *f* is a frictional term describing the interactions between the reorienting entity and its immediate environment,^39^ *k*_B_ is the Boltzmann constant, *T* is the temperature of the system and *S* is a shape factor to account for the ellipsoidal shape of the rotor.^40^

As noted previously, there are numerous reports on both the existence and extent of the decoupling of dynamics over different length scales in DES systems.^30,32,42^ These reports have shed light on the existence of spatial heterogeneity in these systems which, due to the observed variability in decoupling for different systems, has been deemed to be dynamic in nature. The rotational diffusion dynamics of charged probes in DES mixtures can serve as an effective probe of dynamic decoupling within these systems due to the ability of the different chromophores to selectively interact with local environments of different polarity. Decoupling in these systems is manifested as a nonlinear dependence (i.e., *p* ≠ 1 in eq 3) of the measured reorientation time constant, τ_OR_, a quantity reflecting the local environment, on the bulk viscosity.

3 $$\tau_{\text{OR}}\propto {\left(\frac{\eta }{T}\right)}^{p}$$

As implied in eq 2, the expected dependence of τ_OR_ on (η/*T*) is linear (*p* = 1), and deviations from this behavior in a (homogeneous) molecular liquid are taken to be due to changes in the nature of the frictional interaction term, *f*. Systems where *f* = 1 are said to be in the so-called stick-limit, reflecting comparatively strong intermolecular “frictional” interactions.^39^ The stick-limit is typically appropriate for polar, charged chromophores dissolved in polar solvent systems. For weaker frictional interactions that are characteristic of the interactions between nonpolar chromophores and nonpolar solvents, the *f* term is described in the slip-limit. The details of slip limit behavior have been described by Hu and Zwanzig and the value of *f* depends on the effective rotor shape, *S*, calculated according to the Perrin equations. This treatment of the viscosity-dependence has, in general, not been applied to DESs because of their known heterogeneous nature. In Figure 2, we compare our reorientation data for each chromophore to the expected stick- and slip-limit reorientation times.^39,40^

**Figure 2:**
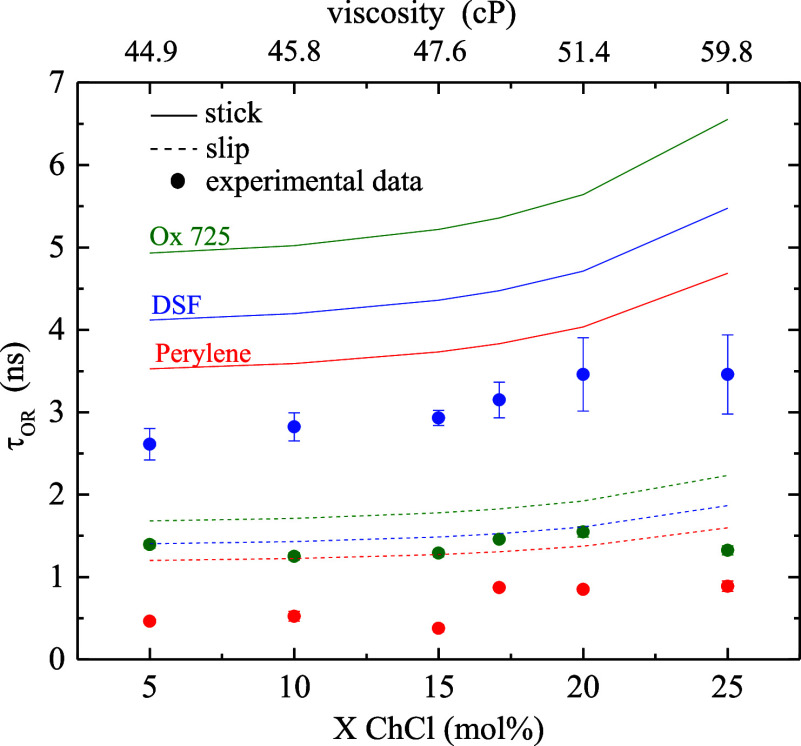
Comparison of experimental τ_OR_ data (points) to calculated predictions of the modified DSE model (eq 2) in the stick and slip limits. Ox725 data and calculations are in green, perylene in red, and DSF in blue. Data points are the average of six measurements for each sample and the uncertainties are ± 1σ.

The rotational diffusion data for each chromophore in the ChCl/1,3-propanediol systems are consistent with our previous findings for the ChCl/ethylene glycol and ChCl/glycerol systems. The dianionic DSF probe exhibits the strongest interactions with the DES system, followed by the Ox725 cation and then neutral perylene. The implication of this finding is that the DES anion is integral to the dynamics and molecular organization of the DES system local environment.^33,34^ The weaker interactions of the cation relative to the anion in these mixtures are consistent with the findings of a molecular dynamics study conducted by Spittle et al.^43^ In that work, the lifetime of the choline-glycerol hydrogen bond was found to be much shorter than that of the chloride (Cl^–^)-glycerol bond, implying weaker interactions between the cationic constituent and the polyol relative to those of the anion. In the vicinity of 15 mol % ChCl, there is an abrupt break in the composition-dependent trend, implying a change in the organization of the local environments of these probes. Unfortunately, detailed structural information on the local environment cannot be elucidated from these data alone.

The expected viscosities of the ChCl/PD DES mixtures were calculated using experimental results from Pandian et al.^44^ and an empirical relation derived by Gajardo-Parra et al. (eq 4).^45^

4 $$\eta =\eta_{0}+A\exp\left(k\left(mol\;\%\;ChCl-x_{0}\right)\right)$$

where η_0_ = 44.20 cP, *A* = 0.333, *x*_0_ = 0 and *k* = 0.154. It is important to note that this function is purely an empirical one and is not intended to reflect a first-principles model of the viscosity composition-dependence. This equation exists for the purpose of extrapolating the viscosities of compositions of ChCl/PD, with the understanding that viscosity is a continuous function of composition. Keeping these issues in mind, we calculated the respective stick and slip limits of these mixtures according to the modified DSE model of eq 2.

For DESs, a deviation from either stick or slip boundary conditions indicates decoupling of local viscosity from that of the bulk system. Viscosity decoupling is a consequence of dynamic heterogeneity, which we have observed in both the ChCl/EG and ChCl/Gly systems.^33,34^ For the ChCl/PD system, we observe prominent decoupling for all three chromophores. This finding, together with the data in Figure 2, suggests that all probe environments exhibit a distinct microscopic structural organization compared to the bulk. The prominence of this effect in the ChCl/PD system implies that the relevant domain sizes of the dynamic heterogeneities are substantially larger than the molecular length scale sensed by the rotational diffusion measurements.

In light of this finding, it is useful to determine whether there are any composition-dependent changes in the dielectric response of the chromophore local environments, and this property can be inferred from fluorescence lifetime data.^46,47^ That is, it is well-known that changes in the dielectric response of the immediate environment of chromophores will affect fluorescence lifetimes. Fluorescence lifetimes may also be affected by nonradiative decay pathways originating from intermolecular interactions with chromophores. To address these issues and their potential impact on our rotational findings, we report measured fluorescence lifetimes of the chromophores in the ChCl/PD system in Figure 3.

**Figure 3:**
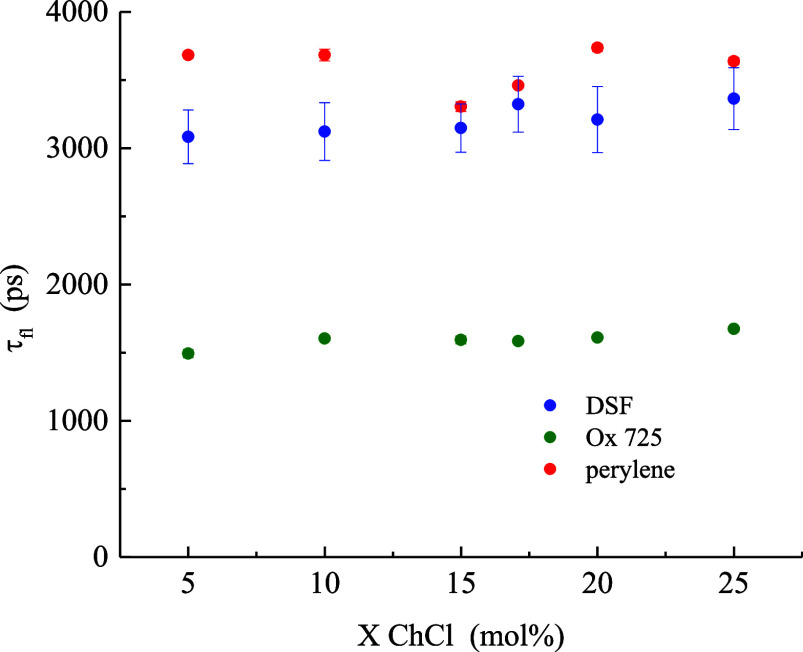
Fluorescence lifetimes of Ox725 (green), perylene (red) and DSF (blue) as a function of DES composition. Data points are the average of six measurements for each sample and the uncertainties are ± 1σ.

For each chromophore, variations in excited-state lifetime with DES system composition are minimal. That is, chromophores that displayed the strongest interactions with the DES systems (DSF, Ox725) based on rotational diffusion behavior show only minor composition-dependent variations in lifetime. All fluorescence lifetimes decayed as single exponentials, as expected. From these data, we cannot discern any significant composition-dependent variation in the dielectric response of the DES system. This finding implies that each chromophore is localized within a relatively composition-invariant environment, consistent with the characteristic length scale of dynamic heterogeneity within the ChCl/PD DES system being much larger than the distance the chromophore can translate during its excited-state lifetime. We estimate that distance to be ∼3 Å based on the known relationship between rotational and translational diffusion constants.

To understand the dependence of DES local organization on HBD properties, it is instructive to compare the experimental data for the different HBDs shown in Figure 1. Since type III DESs, specifically ChCl/X mixtures, are inexpensive, nontoxic or of comparatively low toxicity, and most have been studied extensively, these systems are ideal for elucidating common trends originating from systematic variations in the HBD constituent. To discern the effects of −OH spacing and the effects of elongating the alkyl chain of HBDs on this type of DES, we consider separately the issues of the number of −OH groups per polyol molecule and the −OH group spacing in the polyol.

By comparing the structures of the HBDs (Figure 1), several interesting trends emerge. First and foremost, all three HBDs display significantly stronger association with the dianion probe, followed by the monocation, with the weakest association observed for the neutral, nonpolar probe perylene. This is not unexpected and is consistent with previous findings that report on the prominent role of the chloride ion in mediating the dynamics and structural organization of ChCl-containing DESs at a molecular level.^32,33,48^ For the ChCl/PD composition-dependent data (Figure 2), there is a change in dynamics seen at X ChCl ∼ 15 mol %, manifested most prominently through the anion chromophore dynamics. This trend is likewise observed for the ChCl/EG and ChCl/Gly DESs, also at ∼15 mol % (Figures 4 and 5). These data highlight the importance of ChCl in mediating organization and dynamics within these systems. We first compare the experimental data as a function of the number of −OH groups per polyol, and then consider the role of −OH group spacing.

**Figure 4:**
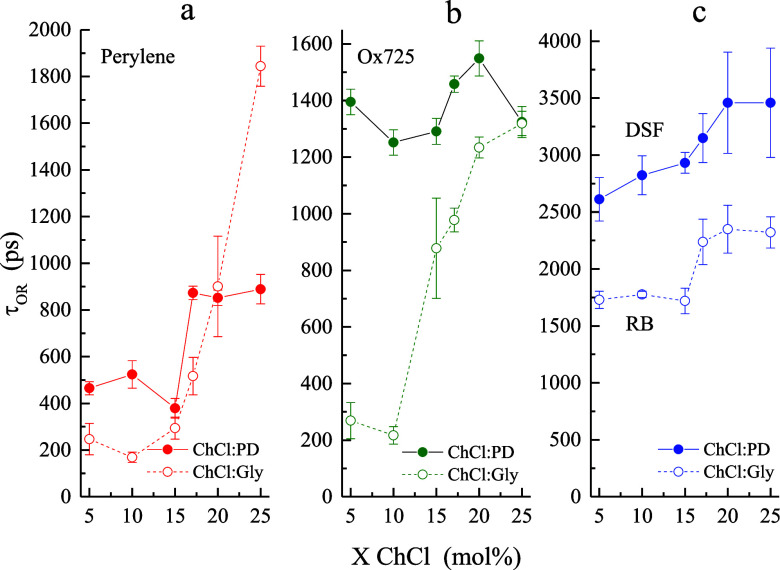
Comparison of τ_OR_ data as a function of DES composition between 1,3-propanediol and glycerol constituents for the four chromophores used: (a) perylene, (b) Ox725, and (c) DSF and RB. The dashed line indicates ChCl/Gly and the solid line denotes ChCl/PD data. Rose Bengal (RB) was used as the dianion probe in ChCl/Gly studies. Data points are the average of six measurements for each sample and the uncertainties are ± 1σ.

**Figure 5:**
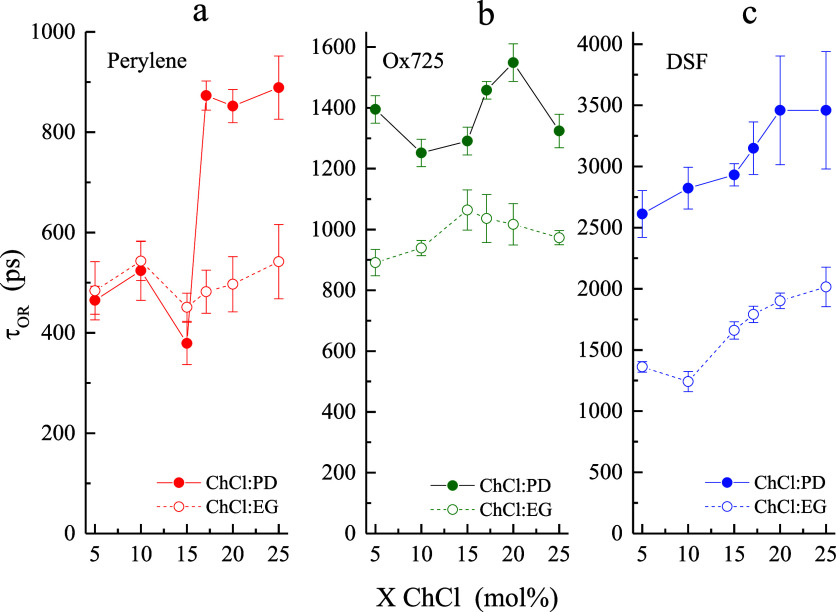
Comparison of τ_OR_ data as a function of DES composition between 1,3-propanediol and ethylene glycol for the three chromophores used: (a) perylene, (b) Ox725, and (c) DSF. Dashed lines indicate ChCl/EG data and solid lines indicate ChCl/PD data. Data points are the average of six measurements for each sample and the uncertainties are ± 1σ.

In comparing the reorientation data for each chromophore in Figure 4, it is important to note that the bulk viscosities at room temperature of 1,3-propanediol and glycerol differ by a factor of ca. 30 (∼50 cP vs ∼1400 cP). Despite this difference, none of the τ_OR_ values differ by more than a factor of 5 for a given ChCl mole fraction, and in most instances are well within a factor of 2. This observation underscores the significant decoupling that exists between molecular-scale (“microviscosity”) and macroscopic domains within these DESs. Comparing the effects of two polyols with different numbers of −OH groups for the charged chromophores, the interactions between the chromophores and ChCl/PD DESs are uniformly stronger than the interactions with ChCl/Gly DESs. This finding suggests that the triol glycerol is more self-associative than the diol 1,3-propanediol, which is consistent with expectations on energetic grounds. When examining the reorientation of the dianionic chromophores, the comparison is all the more pronounced (Figure 4c). The reorientation time constant for DSF in ChCl/PD DESs is longer than for Rose Bengal (RB) in ChCl/Gly DESs, despite the larger hydrodynamic volume of RB (432 Å^3^ vs 264 Å^3^). It appears that the 1,3-propanediol interacts more strongly with the DSF chromophore than glycerol interacts with RB. It is possible that the halogen functionalities on RB play a steric role, but this structural difference cannot account for the expected effect of the viscosity differences of the two polyol-based DESs. For the monocation, we likewise observe less interaction between the probe and glycerol relative to that of 1,3-propanediol (Figure 4b). This finding is consistent with the DES system interactions with the dianions and is likely accounted for by the prominent self-association of glycerol. In both instances, these findings are consistent with significant compositional heterogeneity in these DESs. Work by Faraone et al. shows that in ChCl/glycerol mixtures, the bulk of the microstructure is mediated by Gly–Gly interactions, with ChCl serving primarily as a plasticizer.^49^ This network has been reportedly destabilized on the addition of ChCl with noticeable effects on reaching 10% mol ChCl in ChCl/glycerol mixtures.^43,50^ Therefore, it is likely that above 15 mol % ChCl the hydrogen bonding network is disrupted, and more hydrogen bonding occurs between the Cl^–^ anion and glycerol than between choline and glycerol. 1,3-Propanediol is known to self-associate but given the higher degree of interaction between the charged probes and the solvent relative to that of glycerol, it is likely that this network is not as robust as that of glycerol.^51^ It is likely that the presence of a greater number of −OH groups may create additional opportunities for polyol-based HBD constituents to engage in self-association.

The behavior of the neutral probe perylene is especially interesting as we observe similar trends below the 15 mol % ChCl composition for both DESs. For higher mole fractions of ChCl, perylene associates much more strongly with glycerol than with 1,3-propanediol. A potential explanation for this could be that as the hydrogen bonding network is disrupted, glycerol molecules are more free to interact with perylene molecules. It is noteworthy that the approximate spacing between glycerol −OH groups (∼2.5 Å) permits interaction between a single glycerol molecule and perylene (width ≈ 5.6 Å). Perylene has been known to coordinate water and simple alcohols via π-bonding to the outer rings of the complex.^52^ Thus, a similar phenomenon may be occurring between glycerol and perylene following the disruption of the hydrogen-bonding network.

We consider next the effect of −OH group spacing for the two diols studied, ethylene glycol and 1,3-propanediol. We show comparisons of data for ChCl/PD and ChCl/EG DESs for the three chromophores used in this work (Figure 5). Although the bulk viscosities of 1,3-propanediol and ethylene glycol differ by approximately a factor of 2.5, τ_OR_ values for a given fluorescent probe at a particular composition (mol % ChCl) show less than a 2-fold variation between the ChCl/EG and ChCl/PD systems. As noted above for the ChCl/PD and ChCl/Gly comparison in Figure 4, the DES-chromophore interactions were strongest for the dianionic chromophore, and that trend is also seen for the comparison of ChCl/PD and ChCl/EG data in Figure 5. The cationic Ox725 and neutral perylene chromophores interact less strongly with the DES systems, which is consistent with all type III systems studied so far.^33,34^ For both Ox725 and DSF, the reorientation time constants in the ChCl/PD DESs are not quite twice what is seen in the corresponding ChCl/EG DESs, and this difference is at least qualitatively consistent with the difference in the bulk viscosities of these two diols. The two diols are of similar polarity; 1,3-propanediol is reported to have a dipole moment of 2.6 D, whereas ethylene glycol is reported to have a dipole moment of 2.4 D, although the actual dipole moment will be dependent on the (dynamic) conformation of the molecule.^53,54^ Interestingly, Burda and colleagues reported that ethylene glycol, while capable of forming an extensive hydrogen bonding network, rapidly forms and breaks hydrogen bonds.^44^ In the same study, the lifetimes of the hydrogen bonds of 1,3-propanediol were determined to be much longer, implying somewhat stronger bonds.^44^ Additionally, previous MD studies have predicted a highly ordered supramolecular structure for neat ethylene glycol which is tightly packed. The same study determined that the supramolecular structure of neat 1,3-propanediol was less densely packed.^55^ These findings suggest that both the characteristic domain size and the lifetime of mesoscopic heterogeneities in these DES systems differs in a manner that is mediated by the HBD. It is also worthy of noting that the disruption of the H-bonded HBD network by ChCl is seen more prominently for the ChCl/PD DES, and this observation may speak to the characteristic domain sizes of the two DES systems.

It is instructive to consider the relative hydrogen bonding energies for the two diols. Agayan, Balabaev and Rodnikova reported the (calculated) hydrogen bond lifetimes for ethylene glycol and 1,3-propanediol intermolecular hydrogen bonds to be 18 and 23 ps, respectively.^51^ Using eq 5, and the assumption that the energy of a hydrogen bond is approximately 5.5 kcal/mol,^51,56^ the Arrhenius prefactor (*A*) was calculated for each constituent and determined to be 5.47 × 10^14^ s^–1^ for PD and 7.03 × 10^14^ s^–1^ for EG.

5 $$k=\tau^{-1}=A\exp\left(-\Delta E/RT\right)$$

Using the respective computed prefactors, bond energies for the OH–Cl^–^ bond in the ChCl/EG and ChCl/PD systems were calculated using results from Pandian et al.^44^ Bond energies in the ChCl/PD system range from 7.5–7.6 kcal/mol, and energies in the ChCl/EG system range from 7.1–7.4 kcal/mol. Respective uncertainties were calculated from reported values and we have determined that these values are statistically different (Tables S2 and S3). This energetic difference between these constituents could potentially be explained by van der Waals (vdW) interactions or greater conformational freedom for 1,3-propanediol. The magnitude of vdW interactions is thought to be on the order of 0.2–0.5 kcal/mol for alcohols, which would quantitatively address the calculated energetic differences between these mixtures.^57^ Considering that the OH–Cl^–^ bonds are ca. 2 kcal/mol greater than that of the hydrogen bond, it is likely that the OH–Cl^–^ interactions dominate in both of these systems. This could explain the high degree of interaction that we observe in the dianion probe relative to its cationic and neutral counterparts.

In the case of the neutral chromophore, it is possible that a phenomenon similar to what we observe in the case of ChCl/Gly systems occurs. Pandian et al. report that PD–PD interactions decrease as a function of increasing mol % ChCl.^44^ It is therefore possible that the −OH moieties of 1,3-propanediol undergo bonding to perylene π-system in a manner similar to that observed for ethanol.^52^ Taking all of the above observations into account, we conclude that wider spacing between −OH groups results in longer-range intermolecular interactions purely on structural grounds, leading to somewhat stronger hydrogen bonding events and more intermolecular interactions, resulting in increased effective local viscosity (i.e., microviscosity). In this context, 1,4-butanediol will serve as an ideal testbed to strengthen this hypothesis.

## Conclusions

In keeping with our previous results reported for ChCl/polyol systems, we have determined that ChCl/PD mixtures show significant viscosity decoupling between local environments and that of the bulk, consistent with pronounced dynamic heterogeneity. It is also important to note that our results are consistent with an optimal eutectic mixture of ChCl/PD at approximately 15 mol % ChCl, in agreement with results by Sangoro and co-workers.^58^ On comparing DESs comprising HBDs of varying alkyl chain length and number of −OH groups (ethylene glycol, glycerol, and 1,3-propanediol), it is interesting to note that, in all cases, a structural perturbation sensed at the molecular level occurs in the DESs in the vicinity of 15 mol % ChCl. This result may imply that the optimal eutectic point is compositionally remarkably constant across DESs comprising these different polyol HBDs, a finding that merits further investigation, and if that proves to be the case, the implication is that the pronounced freezing point depression seen for this class of DESs is mediated primarily by the hydrogen-bonded network within them.

Comparing the results of this study with previous work allows for the possible elucidation of the effects of the number of −OH groups and −OH group spacing on the microenvironments within these DESs. The examination of 1,3-propanediol and glycerol reveals interesting results concerning the influence of the number of −OH groups on the polyol HBD component in ChCl/HBD mixtures. We posit that with larger numbers of −OH groups, strong hydrogen bond networks form, leading to self-association, higher viscosities, and substantial viscosity decoupling of the local environments formed by these systems. These networks appear to be disrupted at ca. 15 mol % ChCl (i.e., a ChCl/PD molar ratio of nearly 1:6). Our comparison of ethylene glycol and 1,3-propanediol-containing DESs indicates that, with greater −OH spacing, the relative importance of hydrogen bonding increases, leading to stronger overall interactions with charged constituents (i.e., Cl^–^ anions) and a higher overall microviscosity. Understanding how to design or modulate dynamic heterogeneity within DESs is crucial for applications such as catalysis and electrochemistry, for example. Local variations in viscosity, polarity, and ion mobility can create microenvironments that either enhance or inhibit reaction rates and selectivity, thereby affecting yields and efficiency in catalytic processes, or significantly influence charge transfer rates, electrodeposition quality, and overall device performance in electrochemical systems.

Finally, it is notable that our data reveal a significant shift in probe response near 15 mol %, suggesting that the formation of a robust hydrogen-bonded network characteristic of DESs may occur at much lower ChCl compositions than traditionally anticipated for the canonical ChCl/HBD system. This finding reinforces a growing body of literature indicating that the definition of a DES should not be limited to a specific mole fraction ratio, but rather grounded in the emergence of distinct microstructural and physicochemical properties. These results highlight the increasingly nuanced nature of deep eutectic behavior and underscore the importance of connecting observable macroscopic properties to underlying molecular-level interactions. Our findings align with recent discussions in the field and contribute meaningfully to the ongoing debate, prompting further inquiry and careful reconsideration of the compositional thresholds that define DESs—a perspective that is both timely and important given their rapidly expanding range of applications.

## Supplementary Material



## Notes

Details on DES preparation, reference data for hydrogen-bond lifetimes and OH–Cl^–^ bond strengths in the studied DESs, instrument response functions, and representative fluorescence lifetime and anisotropy decay data (PDF)
