# Supplementary material for: The Importance of Hydrogen Bonded Networks in the Dynamic Heterogeneity of Deep Eutectic Solvents
Source: J Phys Chem B. 2025 Jun 16;129(25):6300–8. doi: 10.1021/acs.jpcb.5c02468 (PMC12207578; doi:10.1021/acs.jpcb.5c02468)
Supplement: Supplementary file 1 [file jp5c02468_si_001.pdf]

The Importance of Hydrogen Bonded Networks in the Dynamic Heterogeneity of Deep Eutectic Solvents

Allison Stettler,[1] Gary A. Baker[2] and G. J. Blanchard[1,*]

[1] Michigan State University, Department of Chemistry, East Lansing, MI 48824-1322 USA
[2] University of Missouri-Columbia, Department of Chemistry, Columbia, MO 65211 USA

Supporting Information

Table S1: Summary of ChCl:PD DES preparation.

Table S2: Reference data from Pandian *et al.* (τ) and calculated energies of OH-Cl^- bonds for 1,3-propanediol as a function of mol% ChCl.

Table S3: Reference data from Pandian *et al.* (τ) and calculated energies of OH-Cl^- bonds for ethylene glycol as a function of mol% ChCl.

Figure S1: Instrument response functions shown alongside respective chromophore lifetime decays for 1:3 ChCl:PD.

Figure S2: Calculated anisotropy decays (R(t)) for each chromophore with best-fit decays in 1:3 ChCl:PD.

Figure S3: Calculated fluorescence lifetimes (I(t)) for each chromophore with best-fit decays in 1:3 ChCl:PD.

[*] Author to whom correspondence should be addressed. Email: blanchard@chemistry.msu.edu, Tel: +1 517 353 1105.

Table S1: Summary of ChCl:PD DES preparation.

X ChCl (mol%)	ChCl:PD molar ratio	mass ChCl (g)	mass PD (g)
5.00_1	$1:18.99_6$	2.274	23.541
9.99_7	$1:9.00_3$	4.286	21.030
14.99_6	$1:5.66_8$	6.234	19.258
17.09_1	$1:4.85_1$	6.892	18.221
19.99_9	$1:4.00_0$	8.002	17.445
24.98_9	$1:3.00_2$	9.509	15.556
28.56_2 [a]	$1:2.50_1$	10.814	14.740
33.29_9 [a]	$1:2.00_3$	12.111	13.221

[a] These samples remained as supercooled liquids before eventually solidifying at room temperature and were therefore not studied.

Table S2: Reference data from Pandian *et al.* (**τ**)[1] and computed energies of OH-Cl⁻ bonds (this work) for 1,3-propanediol at varying mol% ChCl. $T = 298$ K

Mol % ChCl	τ (ps)	E_a (kcal/mol)
5	600 ± 100	7.52 ± 0.10
10	560 ± 20	7.48 ± 0.02
15	620 ± 10	7.54 ± 0.01
20	660 ± 70	7.58 ± 0.06
22	630 ± 10	7.55 ± 0.01
25	680 ± 30	7.60 ± 0.03
27	720 ± 80	7.63 ± 0.07
30	770 ± 30	7.67 ± 0.02
33	740 ± 10	7.65 ± 0.01

Table S3: Reference data from Pandian *et al.* (**τ**)[1] and computed energies of OH-Cl⁻ bonds (this work) for ethylene glycol at varying mol% ChCl. $T = 298$ K

Mol % ChCl	τ (ps)	E_a (kcal/mol)
5	240 ± 20	7.13 ± 0.05
10	300 ± 20	7.26 ± 0.04
15	300 ± 10	7.26 ± 0.02
20	320 ± 10	7.30 ± 0.02
22	330 ± 10	7.32 ± 0.02
25	360 ± 10	7.37 ± 0.02
27	400 ± 10	7.43 ± 0.01
30	420 ± 10	7.46 ± 0.01
33	420 ± 20	7.46 ± 0.03

Table S4: Experimental fluorescence lifetimes, zero-time anisotropy and reorientation times for perylene, oxazine 725 and disodium fluorescein as a function of ChCl:PD composition. Values presented are the average of six measurements for each sample with an uncertainty of ± 1σ.

X ChCl (mol %)	Perylene		Oxazine 725		Disodium fluorescein	
	τ_{fl} (ps)	τ_{OR} (ps)	τ_{fl} (ps)	τ_{OR} (ps)	τ_{fl} (ps)	τ_{OR} (ps)
5	3684±23	465±28	1494±30	1395±45	3083±197	2612±191
10	3683±43	524±59	1605±18	1252±45	3122±212	2823±171
15	3305±35	379±42	1594±28	1291±46	3148±177	2931±91
17.1	3461±8	873±29	1585±8	1458±29	3323±205	3149±215
20	3737±26	852±33	1611±7	1549±62	3209±242	3459±444
25	3638±25	889±63	1674±19	1324±55	3364±227	3459±480

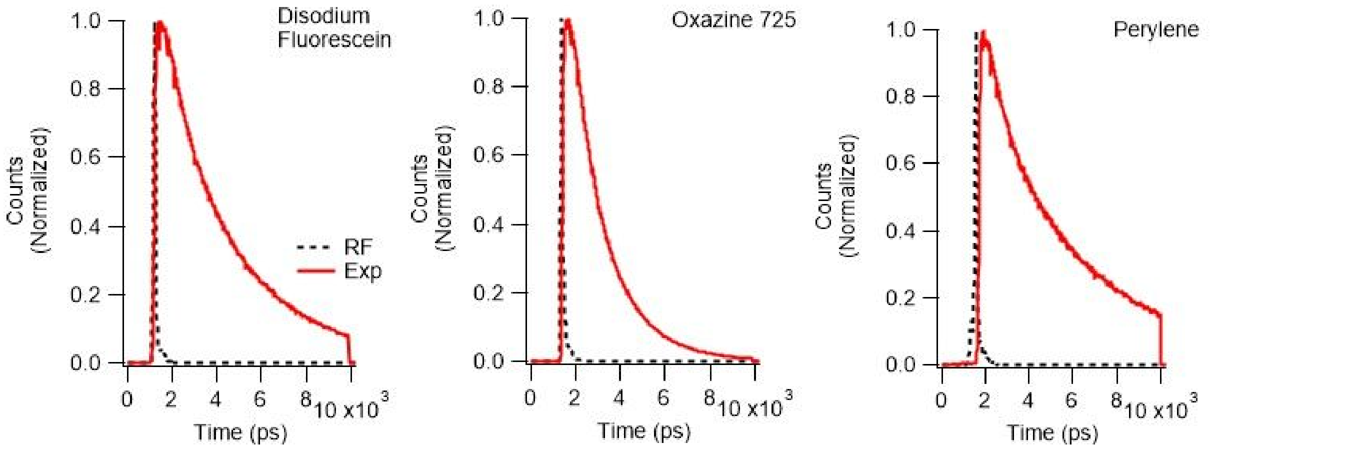

Figure S1. Instrument response functions (RF, black dash) shown with respective chromophore lifetime decays (red) in 1:3 ChCl:PD.

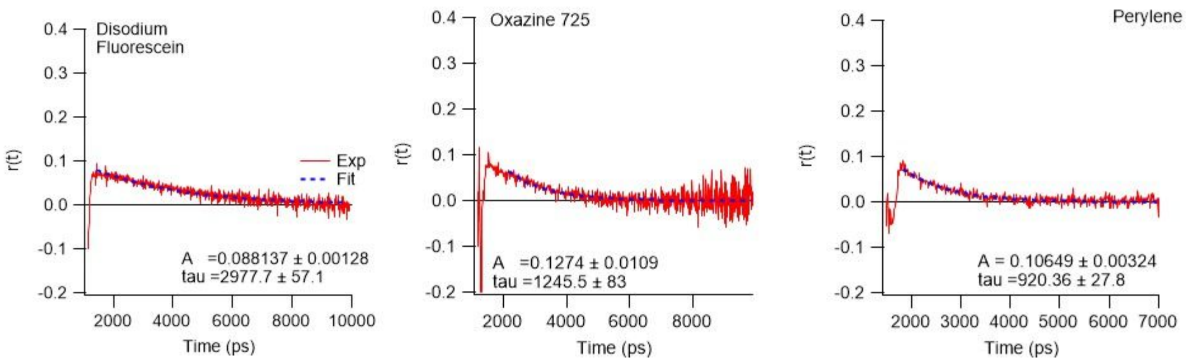

Figure S2. Example calculated anisotropy (red) for each chromophore with fits (blue dash) for 1:3 ChCl:PD.

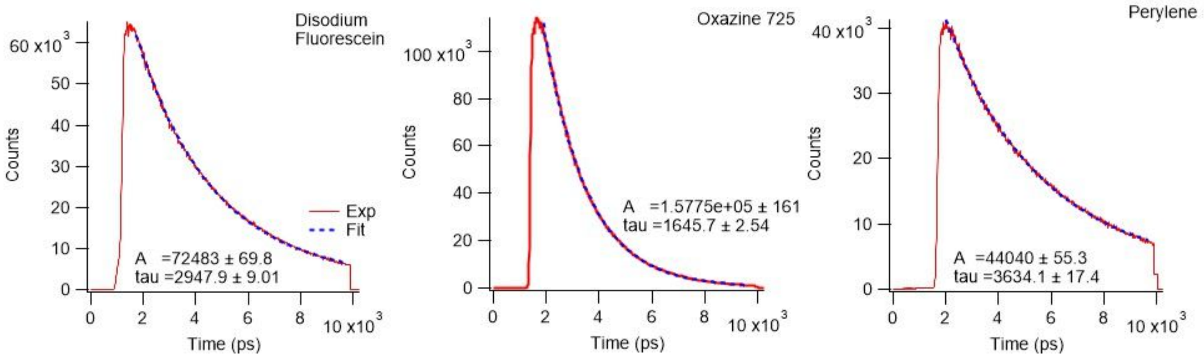

Figure S3. Representative fluorescence intensity decays (red) for each chromophore alongside single-exponential fits (blue dash) in 1:3 ChCl:PD.

Further data available on request.

References

(1) Pandian, R.; Kim, D.; Zhang, Y.; Alfurayj, I.; Prado, D. M.; Maginn, E.; Burda, C. Chain length and OH-spacing effects on diol-based deep eutectic solvents. *J. Mol. Liq.* **2024**, *393*, 123534. DOI: https://doi.org/10.1016/j.molliq.2023.123534.
